# Insights into Fungal Mitochondrial Genomes and Inheritance Based on Current Findings from Yeast-like Fungi

**DOI:** 10.3390/jof10070441

**Published:** 2024-06-21

**Authors:** Jintian Tang, Leilei Zhang, Jinghan Su, Qingwen Ye, Yukang Li, Dinghang Liu, Haifeng Cui, Yafen Zhang, Zihong Ye

**Affiliations:** Zhejiang Provincial Key Laboratory of Biometrology and Inspection & Quarantine, College of Life Sciences, China Jiliang University, Hangzhou 310018, China; jintiantang@cjlu.edu.cn (J.T.);

**Keywords:** fungi, mitochondrial genome, mtDNA, replication strategy, mitochondrial inheritance

## Abstract

The primary functions of mitochondria are to produce energy and participate in the apoptosis of cells, with them being highly conserved among eukaryotes. However, the composition of mitochondrial genomes, mitochondrial DNA (mtDNA) replication, and mitochondrial inheritance varies significantly among animals, plants, and fungi. Especially in fungi, there exists a rich diversity of mitochondrial genomes, as well as various replication and inheritance mechanisms. Therefore, a comprehensive understanding of fungal mitochondria is crucial for unraveling the evolutionary history of mitochondria in eukaryotes. In this review, we have organized existing reports to systematically describe and summarize the composition of yeast-like fungal mitochondrial genomes from three perspectives: mitochondrial genome structure, encoded genes, and mobile elements. We have also provided a systematic overview of the mechanisms in mtDNA replication and mitochondrial inheritance during bisexual mating. Additionally, we have discussed and proposed open questions that require further investigation for clarification.

## 1. Introduction

Mitochondria are crucial organelles in eukaryotic organisms, playing roles in the synthesis of cellular energy, the maintenance of membrane structure dynamics, and the regulation of apoptosis, among other functions [1]. Mitochondria are semi-autonomous organelles that contain their own set of genetic information, known as the mitochondrial genome. The mitochondrial genome varies significantly among different eukaryotic organisms. The endosymbiotic theory suggests that mitochondria originate from a common ancestor with the incorporation of an endosymbiotic alpha-proteobacterium into a host cell [2]. However, as mitochondria have evolved, mitochondrial genomes have diverged significantly among the major groups of eukaryotes. In animals, the mitochondrial genome is a closed circular DNA molecule, encoding a small number of genes related to energy production. It lacks intergenic regions and introns, and its gene arrangement is conserved throughout evolution [3,4]. The mitochondrial genome in plants mostly exists in the form of closed circular DNA molecules, although a small fraction is present as linear DNA molecules. In addition to encoding a large number of genes related to energy production, the plant mitochondrial genome also codes for some plant-specific genes. It possesses intergenic regions and a variable number of introns, making it more complex compared to the mitochondrial genome in animals (Table 1) [4]. Fungi represent a unique kingdom of organisms, closely related to animals and more distantly related to plants [5]. However, the configuration and composition of the mitochondrial genome in fungi are more similar to that in plant mitochondria than in animals. Linear and circular molecules of mitochondrial genomes also exist in fungi [6] There are significant variations in the size of mitochondrial genomes among different fungal species. To date, the smallest fungal mitochondrial genome discovered is 11,198 bp (*Hanseniaspora guilliermondii*), while the largest is 343,690 bp (*Malassezia furfur*) (Table 2). These differences in the number and arrangement of core genes, the quantity and types of introns, and the sequences of intergenic regions contribute to the diversity in the size and structure of fungal mitochondrial genomes. Similar to the structural diversity of mitochondrial genomes, the replication strategies of fungal mtDNA are also complex and are different from those of mammals [7,8]. Fungi also exhibit mitochondrial inheritance during bisexual mating. In the progeny cells of sexual reproduction, mitochondria primarily originate from one parent to avoid cellular heteroplasmy. While the mechanism of mitochondrial inheritance varies among different fungal species, current studies indicate that most mitochondrial inheritance in fungi is tightly regulated and often associated with their mating type loci [9]. In this review, we would like to mainly provide a summary of the constitution of yeast-like fungal mitochondrial genomes, the replication strategies of fungal mtDNA, and the mechanisms of fungal mitochondrial inheritance during bisexual mating, which will provide a reference for the systematic study of fungal mitochondrial genomes.

## 2. Fungal Mitochondrial Genome Structure

Fungal mitochondrial genomes are variable-sized double-stranded DNA molecules, commonly existing in circular or linear form [10]. *Saccharomyces cerevisiae* was the first fungus reported to have a circular mitochondrial genome [11]. Subsequently, a linear mitochondrial genome was revealed in *Hansenula mrakii* [12]. Additionally, the occurrence of coexisting circular and linear forms of mitochondrial genomes in the same fungal species has been demonstrated through restriction enzyme mapping analysis [13].

A circular mitochondrial genome is formed by the fusion of both ends of a linear mitochondrial genome that lacks telomeric loops (t-loops) [13]. These t-loops are recombinant basic units of repetitive sequences that can prevent the formation of circular mitochondrial genomes [14]. In fungi, a notable feature distinguishing the linear mitochondrial genome from the circular mitochondrial genome is the presence of a terminal inverted repeat sequence (TIR) or one to six open reading frames (uORFs) that may potentially generate DNA or RNA polymerases [15]. Studies have revealed that the linear mitochondrial genome of *Batrachochytrium dendrobatidis* consists of three linear segments, each possessing TIRs and containing a uORF that encodes a homolog of the DNA polymerase type B (*dpoB*) [16]. In *Synchytrium*, the mitochondrial genome of *S. endobioticum* is linear, whereas that of *S. microbalum* is circular [17]. Nucleic acid sequence analysis has shown that a *dpoB* homolog exists in the uORF of the *S. endobioticum* mitochondrial genome, and both ends of its mitochondrial genome contain TIRs [17]. In addition, the mitochondrial genome of *S. microbalum* is one-third that of *S. endobioticum* [17]. However, among the 37 linear mitochondrial genomes, only seven have been found to have the presence of *dpoB* homologs, indicating that *dpoB* homologs are not essential in all linear mitochondrial genomes [18]. Interestingly, studies on *Candida parapsilosis* have revealed the existence of linear or circular mitochondrial genomes in strains from different groups [13]. Moreover, the mitochondrial telomeric repeat unit is found in most of the linear mitochondrial genomes in *C. parapsilosis* strains [13].

Therefore, t-loops, TIRs, and *dpoB* homologs may all contribute to the mitochondrial genome adopting either a linear or circular structure, whereas the mechanism still needs further study. In addition to research on the mechanisms and genetic evolution of linear and circular mitochondrial genome formation, studies on *C. orthopsilosis* and *C. metapsilosis* revealed that strains containing linear mtDNA grow faster in YPD medium and form colonies of a certain size earlier compared to strains with circular mtDNA, which shows that strains with linear mitochondrial genomes exhibit a greater adaptive capacity to the growth environment [19]. Thus, the biological functions of linear and circular mitochondrial genomes in fungi are also worthy of in-depth investigation.

## 3. The Coding Genes of the Fungal Mitochondrial Genome

The fungal mitochondrial genome contains 14 conserved core genes, including NADH dehydrogenase subunit (Complex I) genes (*nad1*-*nad6* and *nad4L*), Complex III and IV subunit genes involved in the respiratory chain (*cob*, *cox1*, *cox2*, and *cox3*), and ATP synthase genes (*atp6*, *atp8*, and *atp9*). It also includes small and large subunit rRNA genes (*rns* and *rnl*) and an unknown number of tRNA genes [20,21,22].

However, not all fungal mitochondrial genomes contain these 14 core genes. In terms of the mitochondrial respiratory chain, Complex I is absent in the mitochondrial genomes of most types of yeast, suggesting a possible correlation with the facultative anaerobic respiratory mode in yeast [23]. Nevertheless, multiple alternative NAD(P)H dehydrogenases, which can transfer electrons bypassing complex I, have been identified in the nuclear genomes of *S. cerevisiae*, *C. glabrata*, *Kluyveromyces lactis*, *Eremothecium gossypii*, and *S. pombe* that lack Complex I [24,25]. Moreover, the additional alternative oxidases (AOX), which can transfer electrons from ubiquinone to O_2_ bypassing Complex III and IV, have been identified in the nuclear genomes of various phytopathogens, including *Botrytis cinerea*, *Magnaporthe oryzae*, *Ustilago maydis*, *Sporisorium reilianum*, and the like. And the AOX is mainly responsible for the environmental stresses in fungi [24,26,27,28]. In terms of the ATP synthase gene, studies have found that in the mitochondrial genome of *Erysiphe necator*, there is a significant loss of amino acid sequences in the conserved functional domains of the N-terminal region in *atp9* [29]. This loss results in the functional defect of *atp9*. However, a homolog of *atp9* has been found in the nuclear genome of *E. necator*. It is speculated that this gene might have undergone migration between the mitochondrial and nuclear genomes. The loss of function in mitochondrial *atp9* could potentially be compensated for by the homolog present in the nuclear genome [29]. Moreover, artificially nuclear expressing *atp9* in a mitochondrial *atp9* gene deletion mutant of yeast can lead to its phosphorylation function being achieved, but it may also trigger a stress response in the cell [30]. This suggests that the function of *atp9* is strictly regulated in fungi. Surprisingly, *atp9* has neither been identified in the mitochondrial genome nor the nuclear genome of *Ophiostoma himal-ulmi* [31]. In addition to the absence of *atp9*, *atp8* is also missing in *Stemphylium lycopersici* [22]. The mechanism of how to compensate for the functional loss of these mitochondrial genes in fungi still requires further study. The small and large subunits of rRNA encoded by the fungal mitochondrial genome mainly participate in the synthesis of mitochondrial proteins. However, in the mitochondrial genome of *Morchella conica*, coding sequences for rRNA subunits (*rnl* and *rns*) have not been identified [32]. While the mitochondrial genomes of most fungi encode more than 20 tRNA genes, only 7–9 tRNA coding sequences were found in the mitochondrial genomes of *Harpochytrium hedenii* and *Hyaloraphidium curvatum*. It is speculated that the tRNAs required for protein synthesis are translated by nuclear sequences and then imported into the mitochondria [33,34].

In fungi, the mitochondrial genome may contain additional atypical genes whose products contribute to ribosome assembly (e.g., *rps3*), the promotion of transcription (N-acetyltransferase), RNA maturation, and intron splicing. The ribosomal protein rps3 is the only one that is encoded by the fungal mitochondrial genome. It plays a regulatory role in protein synthesis, exerting various functions during the translation process. In fungi, the *rps3* homolog *VAR1* was initially discovered in yeast, and later, it was subsequently found in ascomycetes and basidiomycetes [35,36]. The *rps3* homologs can be located either within introns or in intergenic regions. In most fungi of Pezizomycotina, *rps3* is located within the introns of *rnl*. However, in fungi of Saccharomycotina, *rps3* is found in intergenic regions, with some located between *trnS* and *rnl* and others located downstream of *trnM* [37]. In *Allomyces* and *Blastocladiella*, *rps3* is located downstream of *trnF* [38]. However, in Basidiomycota, *rps3* is not located in a conserved syntenic unit but instead scattered throughout the different mitochondrial genomes [37]. In the fungi of *Aspergillus*, *rps3* can be located either within the introns of *rnl* or *nad5* [39]. In *Bipolaris sorokiniana*, two independently existing *rps3* homologs have been identified to be located between *cox3* and *nad1*, with lengths of 399 bp and 555 bp, respectively, indicating the possibility of the simultaneous existence of two *rps3* homologs in the same strain [40]. Furthermore, *rps3* has also been found to exist in the nuclear genome. The protein generated by nuclear *rps3* possesses an N-terminal KH domain involved in rRNA binding and a C-terminal ribosomal protein S3 domain. In contrast, the rps3 protein produced by mitochondrial genome expression lacks the KH domain but retains the conserved S3 domain [37]. The absence, structural variations in position, and sequence diversity of *rps3* in mitochondrial genomes may be related to the evolutionary changes in mitochondrial genomes and functions among different fungi.

N-acetyltransferase can catalyze the transfer of an acetyl group between acetyl coenzyme A and an amine. In the mitochondrial genome of fungi, it can be encoded by open reading frames (ORFs) within introns and also exist independently in intergenic regions. The N-acetyltransferase domain-containing protein with a length of 217 aa was initially discovered in the mitochondrial genome intron in *S. lycopersici* [22]. Furthermore, in *Pestalotiopsis fici,* the encoding of aminotransferase has been found as a standalone aminotransferase gene located in an intergenic region [41,42]. In *Annulohypoxylon stygium*, two putative copies of N-acetyltransferase genes are located downstream of *cox2* and within the ORF of the third intron of *cob* [43]. Phylogenetic analysis of N-acetyltransferase genes has shown that it may originate from the nuclear genome and undergo horizontal transfer from the nucleus to the mitochondrial genome [39]. The mechanisms underlying this phenomenon remain to be further investigated. 

The mitochondrial RNase P (*rnpB*) gene, which is responsible for tRNA cleavage, was identified in the fungal mitochondrial genome. The presence of *rnpB* has been identified in some species of basidiomycetes, including *Microbotryum lychnidis-dioicae*, *Schizophyllum commune*, and *U. maydis* [42]. Additionally, the copy number of *rnpB* varies among different fungal mitochondrial genomes, with *M. conica* identified to have two independently existing *rnpB* [32,44]. However, the occurrence of *rnpB* in fungal mitochondrial genomes is generally low. Phylogenetic and RNA secondary structures analyses have shown that the mitochondrial *rnpB* homologs have been lost several times independently in fungi [45].

## 4. Mobile Elements in the Fungal Mitochondrial Genome

Mobile elements are a type of DNA sequence capable of altering their position within a genome or inserting into another genome [46]. One of the widely present mobile elements in the fungal mitochondrial genome is the self-splicing intron. Introns are distributed across various fungal mitochondrial genes, with *cox1* being the primary intron reservoir, followed by *rrnl*, *cox2*, *cob*, *cox3*, and *nad2* [47]. Generally, based on the RNA secondary structure, mitochondrial introns in fungi are typically classified into Group I and II. 

Group I introns primarily consist of 10 conserved helices with a structurally conserved catalytic core, crucial for self-splicing [48]. There are two main mechanisms currently recognized for the migration of Group I introns. One is driven by the homing endonucleases (HEs). Based on the conserved amino acid sequences at the active sites involved in enzyme activity, HEs are classified into four families: GIY-YIG, LAGLIDADG, His-Cys, and HNH [49]. Currently, in the fungal mitochondrial genome, genes encoding GIY-YIG (GIY) and LAGLIDADG (LD) endonucleases have been identified [50]. During intron homing, the HE initiates a double-strand break repair, causing cleavage at a specific HE recognition site within the invading gene (approximately 14–45 nucleotides). After cleavage, the gene containing the intron serves as a template to repair the break, and the intron, along with the HE gene, integrates as a single unit into the new host genome [51]. The frequency of horizontal gene transfer induced by HEs varies significantly among fungal populations and is increasingly recognized as a crucial evolutionary process in fungal evolution [50]. The other one involves reverse splicing via an RNA intermediate. Typically, long-distance intron migration is achieved through the reverse splicing pathway. During the reverse splicing process, the internal guide sequence (IGS) within Group I introns recognizes a complementary target region in the 5′ exon sequence of the host gene through 4–6 base pairings. Subsequently, it inserts into the transcribed RNA. Later, the IGS-containing RNA undergoes reverse transcription, and the resulting cDNA undergoes genomic recombination, integrating the Group I intron into the genome [52]. Group I introns can invade both homologous and heterologous sites during this process [53].

Group II introns are always composed of catalytically active intron RNAs and intron-encoded proteins (IEPs), thereby forming an enzyme with self-splicing functionality. Group II introns contain six secondary structure domains (D1-6). D1 is the largest domain serving as a scaffold for the assembly of other domains, capable of recognizing exon sequences. D2 and D3 domains enhance catalytic efficiency, while the D4 domain encodes a multifunctional IEP in certain intron ORFs. D5 and D6 serve as the catalytic center and branch point in the splicing pathway of introns, respectively [54,55,56]. The intron RNA catalyzes its splicing through a transesterification reaction, followed by binding with the IEP protein to form a catalytically active ribonucleoprotein complex. After recognizing the target sequence at the insertion site, the complex cleaves the target DNA through reverse splicing, using the intron RNA as a template to synthesize cDNA. Finally, the insertion of the Group II intron onto the mitochondrial genome is completed through the host’s DNA repair mechanisms [57].

Interestingly, some mitochondrial plasmids, which contain ORFs of reverse transcriptase or DNA/RNA polymerase and exist in the mitochondria of fungi, have the ability to integrate either wholly or partially into the mitochondrial genome of their host, thus becoming a kind of mobile element. Evidence of the transfer of gene fragments and tRNA fragments between mitochondrial plasmids and mitochondrial genomes has been found in some species of *Neurospora*, *Podospora*, *Agaricus*, *Termitomyces*, and *Moniliophthora* [58,59,60,61,62]. Surprisingly, the occurrence of DNA fragment transfer between mitochondrial plasmids and mitochondrial genomes has been found to be associated with fungal growth and aging in *Neurospora* and *Podospora* [63,64].

## 5. The Replication Strategies of Fungal mtDNA

The replication mechanism of fungal mtDNA is mainly studied in yeast, whereas it is less understood than in humans [58,59,60,61,62]. Except for the similar mechanism of RNA-primed replication in humans, yeast has developed alternative ways by coupling recombination and rolling circle replication [63,64]. 

**Figure 1:**
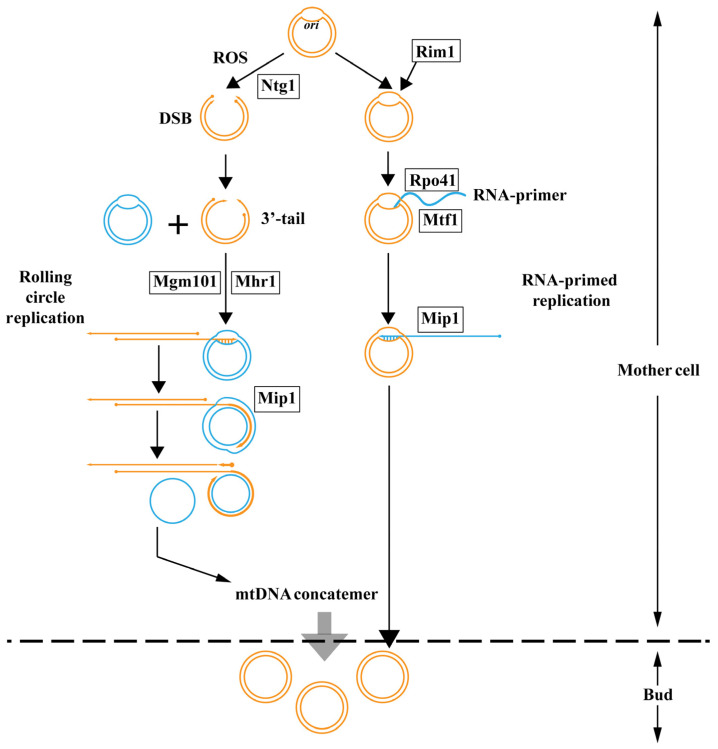
Model of mtDNA replication in yeast. In rolling circle replication (**left**), ROS attack the *ori* region and this leads to a DSB together with Ntg1. The 3′-tail forms subsequently and combines with another *ori* region with the action of Mhr1 and Mgm101, and rolling circle replication proceeds with Mip1. The mtDNA concatemer then moves to the bud. In RNA-primed replication (**right**), single-stranded DNA is stabilized by Rim1.

In mammalian cells, RNA-primed replication initiates from the *rep*/*ori* region [65]. However, sequence analysis shows that *S. cerevisiae* and close relatives have *rep*/*ori*-like elements whereas, no *rep*/*ori*-like elements have been identified in the mitochondrial genome in *C. glabrata* and *H. wingei* [66,67]. Thus, this indicates that the *rep*/*ori*-like elements are not universally present in the mitochondrial genome of yeast. In the process of RNA-primed replication in *S. cerevisiae*, the separated single-stranded DNA is stabilized by a single-strand binding protein, Rim1. RNA polymerase Rpo41 recognizes transcriptional promoters located at *oris* with the help of transcription factor Mtf1 and generates RNA transcripts. These transcripts are subsequently processed to generate primers, thereby leading DNA polymerase γ catalytic subunit Mip1 to proceed with mitochondrial DNA (mtDNA) replication (Figure 1) [65]. Interestingly, the mtDNA can be maintained in the mtDNA defective mutant rho^-^ of *S. cerevisiae*, in which Rpo41 is absent [68]. Therefore, neither *rep*/*ori*-like elements nor Rpo41 are essential for mtDNA replication, which indicates the existence of another mechanism.

The discovery of overlapping replication forks and recombination sites in both *C. albicans* and *C. parapsilosis* has provided increasing evidence that a recombination-mediated rolling circle replication model is a common mode of mtDNA replication in yeast [69,70]. The replication origin on the mitochondrial genome is attacked by reactive oxygen species (ROS), leading to the formation of bubble-like structures. The base damage is ultimately transformed into a double-strand break (DSB) by the base excision repair enzyme Ntg1. The DSB is subsequently processed to create a 3′-tail, which is recognized and bound by the mitochondrial homologous recombinase Mhr1 [71,72]. In addition to Mhr1 mediated recombination, a single-stranded annealing protein Mgm101, which belongs to the Rad52 protein family, has been identified to functionally operate like bacteriophage proteins and catalyze recombination by the single-strand annealing mode [73]. After recombination, the nucleoprotein filament invades a template circular mtDNA molecule, forming a heteroduplex joint and initiating rolling circle replication with Mip1. This process can generate mtDNA concatemers consisting of multiple genome units that are selectively transmitted to growing buds, where concatemers are circularized into monomers (Figure 1). Moreover, single-stranded circles displaced by rolling circle replication can serve as a substrate for single-strand annealing to initiate replication [7,74,75]. Although the recombination-mediated rolling circle replication model has been widely studied and discussed in yeast and is considered the primary mechanism for mtDNA replication, evidence suggests that other mtDNA replication mechanisms also exist in yeast. The diversity of mtDNA replication mechanisms in yeast may be related to its evolution [7]. Furthermore, research on mtDNA replication mechanisms in fungi has mainly focused on yeast, and whether there are more mtDNA replication mechanisms in other fungi remains to be studied.

## 6. The Inheritance of the Fungal Mitochondrial Genome during Bisexual Mating

The inheritance of the mitochondrial genome in fungi during bisexual mating is independent of the nuclear genome and typically exhibits two modes, biparental (BPI) and unpaternal (UPI) inheritance. BPI refers to the inheritance phenomenon where progeny inherit traits from both parents. In *S. cerevisiae* and *Schizosaccharomyces pombe*, the fusion of mating partners can lead to the BPI of the mitochondrial genome and simultaneous recombination of mtDNA [76,77]. UPI is typically associated with inheritance from only one parent, which is a common phenomenon of mitochondrial inheritance observed in many ascomycetes and basidiomycetes. Except for the traditional modes of BPI and UPI in mitochondrial inheritance, some fungal species also exhibit mixed BPI and UPI, with the recombination of mtDNA [78,79,80]. In addition, the mode of mitochondrial inheritance may be different within the same species and can even be influenced by environmental factors [79,81].

For the BPI mode of the mitochondrial genome in *S. cerevisiae* and *S. pombe*, after the fusion of haploid cells following bisexual mating, the nuclei from the two parent cells fuse while the mitochondria remain individually and relatively stable in their positions. Mitochondria near the budding site are then allocated to the progeny cells. The mitochondria in these progeny cells may contain only the mitochondrial genome from one of the parents, or they may contain mitochondrial genomes from both parents, resulting in potentially different mitochondrial genotypes [82,83]. Thus, the position of the budding sites determines whether either or both parents contribute to the mitochondrial genome of progeny cells. Moreover, the progeny cells, which contain mitochondrial genomes of both parents, generate homoplasmic progeny cells after approximately twenty rounds of mitotic cell division [9].

**Figure 2:**
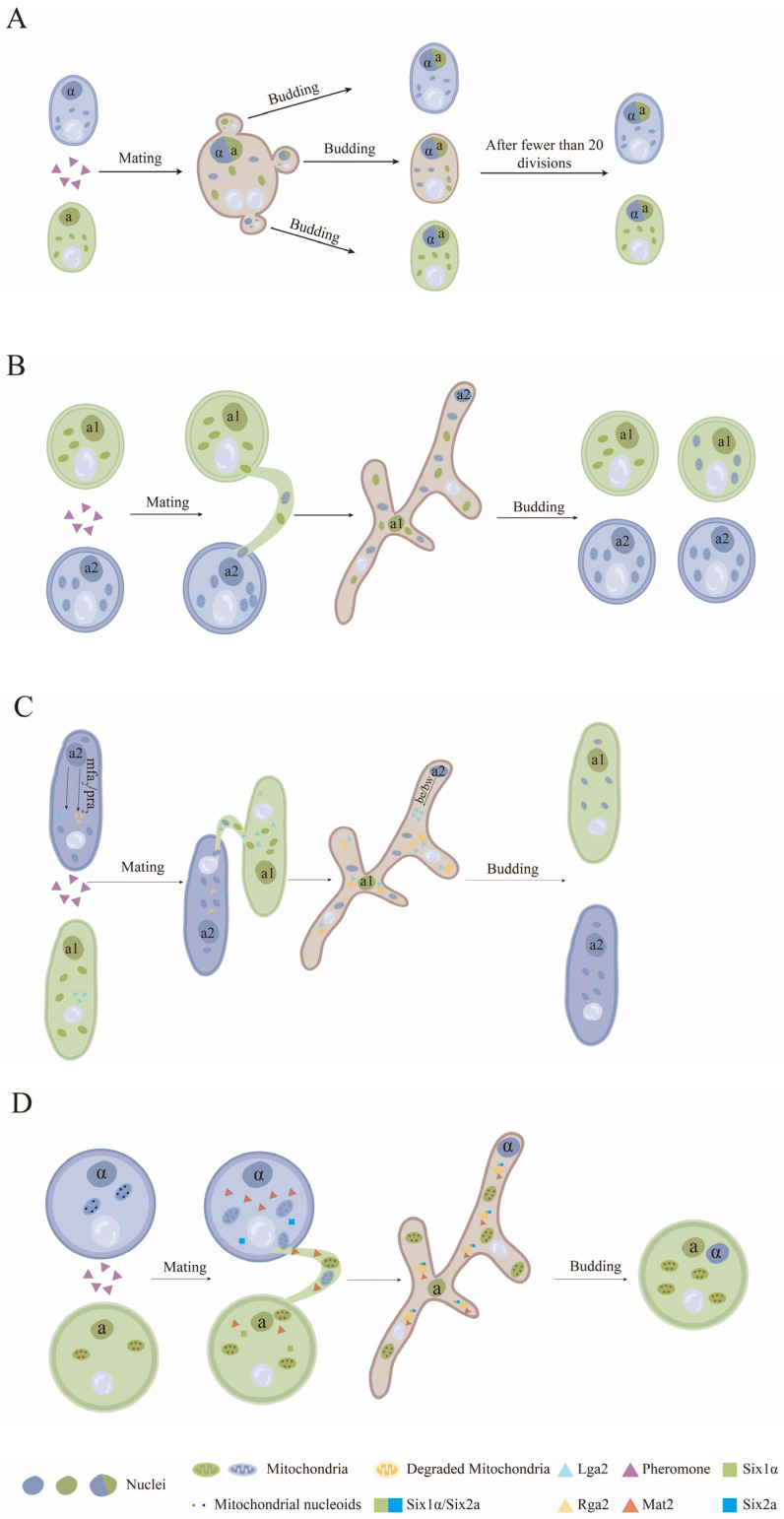
Mitochondrial inheritance model of fungi. (**A**) For the BPI mode in *S. cerevisiae*, the mitochondria are allocated to the progeny cells depending on their positions. (**B**) For the UPI mode, in *M. violaceum*, the mitochondria in the progeny cells of the *a1* mating type are from either the *a1* or *a2* parent, whereas the mitochondria in the progeny cells of the *a2* mating type are almost entirely from the *a2* parental strain. (**C**) In *U. maydis*, Lga2 in the *a2* loci drives the degradation of mitochondria that are from *a1* mating type cells, and Rga2 in the *a2* loci protects the mitochondria that are from *a2* mating type cells. (**D**) In *C. neoformans*, Six2a/Six1α and Mat2 drive the degradation of mitochondrial nucleoids and structures that are from α mating type cells to maintain the UPI of mitochondria. This figure is reproduced from the references of [9,78,88,91,95].

For the UPI mode of the mitochondrial genome, fungi have evolved several different mitochondrial elimination mechanisms that can actively avoid mitochondrial heteroplasmy during bisexual mating [78]. *M. violaceum*, as an obligate pathogen of the Caryophyllaceae, exhibits a heterothallic bipolar mating system, with two forms of mating type loci (*a1* and *a2*). Both forms of *a* locus encode specific pheromone receptors that are homologous to the Ste3 pheromone receptors of *S. cerevisiae* [84]. Upon the fusion of haploid cells of *a1* and *a2* mating types, a conjugation tube forms, through which nuclei and mitochondria are transferred. In the progeny cells with *a1* mating type locus, the frequency of mitochondria from either parent is equal, whereas in the progeny cells with the *a2* mating type, mitochondria are almost entirely (94%) derived from the *a2* parental strain (Figure 2) [85]. However, the mechanism of the UPI in mitochondrial in *M. violaceum* is still unknown.

Bisexual mating of *U. maydis* is regulated by a tetrapolar mating system consisting of the *a* and *b* mating type loci. Genes in a locus encode a precursor for a small lipopeptide pheromone (mfa) and a G-protein-coupled pheromone receptor (pra), while genes in the b locus encode two subunits of a heterodimeric regulatory protein (bE/bW), which is responsible for hyphae growth and virulence [86,87]. There are two different forms (non-homologous) of the *a* mating type locus (*a1* and *a2*) and multiple forms (multiple alleles) of the *b* mating type locus [86]. The *a2* mating type locus contains two ORFs that code *lga2* and *rga2*. The absence of *lga2* promotes the recombination of mtDNA and BPI, whereas the absence of *rga2* favors the inheritance of the mitochondrial genome in strains with the *a1* loci. Studies have shown that the Rga2 protein protects the mitochondria of the *a2* loci strain from degradation by the Lga2 protein [88]. Thus, the *a2* locus has the predominance of mitochondrial genome inheritance in strains. In addition, the common mitochondria degradation pathway, mitophagy, is found to be efficiently triggered by Lga2 in *U. maydis*. However, the lga2-triggered mitophagy is mechanistically distinct from starvation-triggered mitophagy, though the mitophagy triggered by these two different patterns is in all cases dependent on autophagy associated genes *atg8* and *atg11*. Moreover, mitochondrial fission factor Dnm1 (dynamin-like protein) has been verified to participate in the process of mitochondrial fission during mitophagy triggered by Lga2 (Figure 2) [89].

*Cryptococcus neoformans*, as a basidiomycete human fungal pathogen, possesses a bipolar mating system with two mating types, *MATα* and *MATa*. The mating type locus of *C. neoformans* contains genes encoding homeodomain transcription factors (Sxi1α and Sxi2a) as well as genes for pheromones and pheromone receptors [89]. Cells containing opposite mating types (*α* and *a*) undergo bisexual mating under conditions of nutrient starvation. The cell of *MATα* generates a conjugation tube when receiving pheromone signals secreted by the cells of *MATa* nearby [90]. A zygote is formed after cell fusion through the conjugation tube. Meanwhile, the UPI of the mitochondrial genome is strictly controlled, and the mitochondrial genome of hypha germinating from the zygote was determined to come from the *MATa* parent only (Figure 2) [91,92]. A transcription factor Mat2, which is not encoded by the mating type locus, is activated in *MATa* cells and is involved in mitochondrial preservation [91,92]. Thus, overexpression of *Mat2* in the *MATα* strain leads to BPI of the mitochondrial genome while mating with the *MATa* strain in *C. neoformans* [93]. Another set of transcription factors, Sxi1α and Sxi2a, have been identified to form a complex that plays a leading role in the elimination of *MATα* mitochondria by activating unknown downstream factors. Deletion of either *Sxi1α* in the *MATα* strain or *Sxi2a* in the *MATa* strain results in BPI of mitochondrial inheritance in *C. neoformans* [93]. Recently, a negative regulator (*Crg1*) of the pheromone pathway was determined to influence UPI of mitochondrial inheritance in *C. neoformans* under the regulation of *Mat2* [93]. Deletion of *Crg1* in both parents results in BPI of mitochondrial inheritance in progeny cells with the increase of *MATα* mitochondrial genome [94]. A recent study on the observation of mitochondrial elimination process in the zygote of *C. neoformans* showed that the mitochondrial nucleoids and mtDNA of the *MATα* parent are preferentially reduced and then, the mitochondrial structures are degraded, with the remaining mitochondrial nucleoids of the *MATa* parent proliferating during zygote development [95]. Further studies on the mechanism of UPI in *C. neoformans* mitochondrial inheritance have shown that none of the common mitochondrial degradation pathways, including mitophagy, ubiquitination, or methylation, are the key participants involved in *MATα* mitochondrial elimination [94]. However, ATG8-mediated autophagy is responsible for the clearance of mitochondrial structures after the reduction in the nucleoids of the *MATα* parent [95]. Therefore, some unknown mechanisms for mitochondrial degradation are speculated based on the existence of bisexual mating in *C. neoformans*.

## 7. Conclusions

The structure, size, encoded genes, and mobile elements of mitochondrial genomes vary greatly among different fungal species. This diversity may be related to the mitochondrial functions of fungi and their modes of growth, reproduction, and parasitism, which is an intriguing area for further research. Additionally, understanding the relationship between mitochondrial genome differences and genetic evolution in fungal species is crucial for further elucidating fungal phylogenetics. Many unknown mechanisms remain in the replication of mtDNA and mitochondrial inheritance during bisexual mating in fungal mitochondria, which are important for revealing the origin and evolution of mitochondria. This review summarizes existing research on fungal mitochondrial genomes, hoping to provide some assistance to related studies.

## Figures and Tables

**Table 1:** Basic comparison of the mitochondrial genomes of animals, plants, and fungi.

Eukaryotes	Mitochondrial Genome Size	Structure	Characterization of Genes in Mitochondrial Genomes
Animals	10–50 Kb	Circular	Highly conserved set of genes with a few intergenic regions and introns
Plants	60 Kb–12 Mb	Circular (most) or linear (a small amount)	A large number of genes with large intergenic repetitions and a variable number of introns
Fungi	10–350 Kb	Circular or linear	Highly diverse sets of genes with various sequences of intergenic regions and types of introns

**Table 2:** The number and size range of published fungal mitochondrial genomes of four phyla in the kingdom of fungi.

Phylum	Number of Published Fungal Mitochondrial Genomes	Mitochondrial Genome Size (bp)
Ascomycota	693	11,198–272,497
Basidiomycota	403	13,032–343,690
Chytridiomycota	180	19,473–225,604
Zygomycota	30	26,612–83,361

## Data Availability

Data are contained within the article.
